# Food as Medicine for Chronic Disease: A Strategy to Address Non-Compliance

**DOI:** 10.1089/jmf.2020.29007.flg

**Published:** 2020-09-02

**Authors:** Frank L. Greenway

**Affiliations:** Pennington Biomedical Research Center, Baton Rouge, Louisiana, USA.

Rao *et al.* presented a study that is an excellent example of food as medicine.^1^ Food is not only necessary for sustenance, but is also pleasurable, sociable, and an integral part of people's traditional lifestyle. Thus, although medicines may be very advantageous in mitigating the effects of chronic disease, they do not fulfil any of these other characteristics of food. Long-term compliance with medication for chronic disease, despite having efficacy in reducing the burden of chronic diseases, is dismal. Approximately one in five new prescriptions for chronic disease are never filled and 50% are taken incorrectly, in regard to timing, dosing, frequency, and duration, leading to increased disease burden and higher health care costs. Some of the efforts that have been used to improve compliance in taking chronic disease medications include ensuring access to health care providers, educating about the benefits of compliance, reducing medication cost, and enlisting information technology tools. Despite these efforts, compliance is poor.^2^

Rao *et al.* created a wheat-based traditional Indian flat bread called chapati that is an established part of the Indian daily diet. The participants in the trial were given two chapatis twice a day, 6 days a week. By eating these four chapatis, the subjects consumed 4.7 g of *Nigella sativa* and 0.75 g of *Trigonella foenum-graecum* (fenugreek seeds) each of the 6 days per week that the chapatis were consumed. Fenugreek is bitter, especially when toasted, so incorporation into food is limited by taste acceptability. The chapatis used in the study were tested and graded on taste and acceptability as good to excellent. The chapatis were consumed for 12 weeks by 40 subjects who were overweight or obese, 25 of whom had type-2 diabetes. *N. sativa* and fenugreek seeds are common in the Indian diet, and have a reputation of reducing obesity and hyperglycemia. In the first 12 weeks of a weight loss program, people generally lose about two thirds of the weight that may be projected to be lost at the 6-month weight loss plateau. Thus, a 2.89% weight loss from baseline at 12 weeks projects to a 4.34% weight loss at the weight loss plateau. A 5% weight loss is thought to be clinically significant, and a 5% greater weight loss than placebo is one of the criteria to approve a weight loss medication by the U.S. Food and Drug Administration (FDA).

Subjects with diabetes who had baseline hemoglobin A1c (HbA1c) level >7% experienced a mean HbA1c reduction of 0.689% at 12 weeks. Trials to approve medications for the treatment of type-2 diabetes enroll people with HbA1c values >7%, because an HbA1c of <7% is the goal of treatment. A drop of 0.5% or more in HbA1c greater than placebo is the criterion of efficacy used by the U.S. FDA to approve medications for the treatment of type-2 diabetes. Thus, the drop in HbA1c in this study was clinically significant.

Compliance with eating the chapatis during this study was 100%. Although compliance in the context of a clinical trial may differ from compliance during general use in the community, the adherence to the diet intervention in this study is reason for optimism that delivering chronic disease treatments in food will be a step forward in improving compliance. Including treatment for chronic diseases in a traditional food that is eaten daily without changing the taste and preference incorporates tradition and social context into chronic disease treatment in a manner that consuming capsules cannot do.

In summary, this study not only demonstrates a clinically significant improvement in obesity and diabetes, but also incorporates that treatment into a traditional food eaten on a daily basis without compromising its taste or acceptability. The excellent compliance in this trial suggests that incorporating tradition and social context into the delivery of chronic treatments may be a novel and effective way to increase treatment compliance. The use of traditional food as a delivery vehicle for chronic disease treatment may help to solve the expensive public health problem represented by noncompliance with chronic disease treatments.
